# Effect of Different Cantilever Lengths in Polyether Ether Ketone Prosthetic Framework in All-on-Four Technique on Stress Distribution: A Three-Dimensional (3D) Finite Element Analysis

**DOI:** 10.7759/cureus.74544

**Published:** 2024-11-26

**Authors:** Mohammd Almjaddr, Jamal Saker

**Affiliations:** 1 Department of Removable Prosthodontics, Syrian Arab Republic Damascus University Faculty of Dental Medicine, Damascus, SYR

**Keywords:** all on 4, cantilever length, finite element analysis, peek, stress

## Abstract

Background: Determining the distal cantilever length in All-on-Four (All-on-4) implant-supported prostheses is a major factor in the long-term success of these prostheses. The difference in mechanical properties of materials used in the fabrication of these prostheses, such as polyether ether ketone (PEEK), may have an impact on the determination of the cantilever length that best distributes stress.

Aim: To study the distribution of stress in All-on-4 mandibular prostheses in the bone, implants, and framework according to difference cantilever length in PEEK prosthetic framework using three-dimensional finite element analysis.

Materials and methods: A three-dimensional (3D) model of an edentulous mandible was constructed, implants and abutments models were designed by the All-on-4 concept, and two frameworks were constructed from PEEK with different cantilever lengths of 10 and 15 mm. Two study groups were created. Occlusal oblique forces of 600N were applied from the right side at a 45-degree angle, and finite element analysis was performed to obtain the stress distribution in the bone, implants, and framework.

Results: At cantilever length of 10 mm, in the PEEK model, this study found an increase in stress compared to PEEK model at cantilever length of 15 mm in the cortical bone and implants and framework, but PEEK models showed a similar distribution of stress in the spongy bone.

Conclusions: Decreasing the cantilever length in the PEEK model will increase the stress. PEEK models showed deformation of the structure material*.*

## Introduction

Complete edentulism is a dental defect defined as the loss of all permanent teeth in the upper jaw or (and) lower jaw and is considered to have a dangerous effect on nutrition, speech, facial profile, and quality of life in patients with it. The removable complete denture has been the traditional treatment for complete edentulous patients, but there are many common complaints among complete denture patients, including loss of stability and retention of lower dentures due to resorption of the remaining bone with decreased chewing ability as well [1]. Rehabilitation of complete edentulous patients using fixed implant-supported prostheses is a successful and effective treatment option, but in most cases, edentulous patients need bone grafting procedures to place the implants, which increases the cost and risk of complications in addition to the long treatment time [2].

Placing implants in the posterior regions of the mandible is challenging due to the presence of the inferior alveolar nerve canal. Therefore, in order to avoid complex surgical procedures, it has been suggested to place four to six implants between mental foraminas of the mandible as a safe area for implant placement in order to support a fixed screw-retained prosthesis with bilateral distal cantilevers. According to this protocol, when occlusal forces are applied to the distal cantilevers, implants will be subjected to bending forces, which will increase stress on the surrounding bone. Therefore, the All-on-Four (All-on-4) treatment concept features placing two parallel anterior implants and two distally tilted posterior implants in the interforaminal region in order to decrease the cantilever length [2].

Polyether ether ketone (PEEK) is a thermoplastic polymer, a semi-crystalline material that melts at high temperatures. There are many physical properties of this material, such as the modulus of elasticity of 3.6 GPa, which was improved to reach 18 GPa, becoming close to the modulus of elasticity of cortical bone by adding carbon fibers. There are many advantages to this material: no sensitivity to tissue, no metallic taste, and good resistance to abrasion and fracture [3]. PEEK was used in many fields of dentistry, dental implants and implant-supported prostheses due to its similar mechanical and physical properties to bone and dentin [4]. PEEK frameworks have a high fracture and wear resistance, and being shocks absorbent during chewing. Although metals are very strong and durable, patient comfort with the flexibility of this material is major interest [5]. Therefore, PEEK was used in the fabrication of implant-supported frameworks [4,5]. The cantilever acts as an amplifier for the forces applied to the implants, abutment screws, prosthesis screws, adhesive cement, and surrounding bone during mastication. Therefore, determining the appropriate cantilever length is very important because it directly affects the biomechanical stability and long-term success of the prosthesis [6]. There is limited information about biomechanical behavior of different cantilever lengths and its potential effect on stress distribution in the bone around implants, and an appropriate cantilever length has not yet been determined [7].

Most studies have studied the biomechanical behavior of distal cantilever length difference in combination with posterior implant angulation difference without considering the importance of the type of prosthetic framework material on stress distribution [2]. Therefore, Misch recommends that distal cantilever length be determined by the amount of stress applied to the implant system [7]. This study was conducted to obtain the distribution of stress in All-on-4 mandibular prostheses in the bone, implants, and framework according to difference cantilever length in PEEK prosthetic framework using three-dimensional finite element analysis.

## Materials and methods

**Figure 1:**
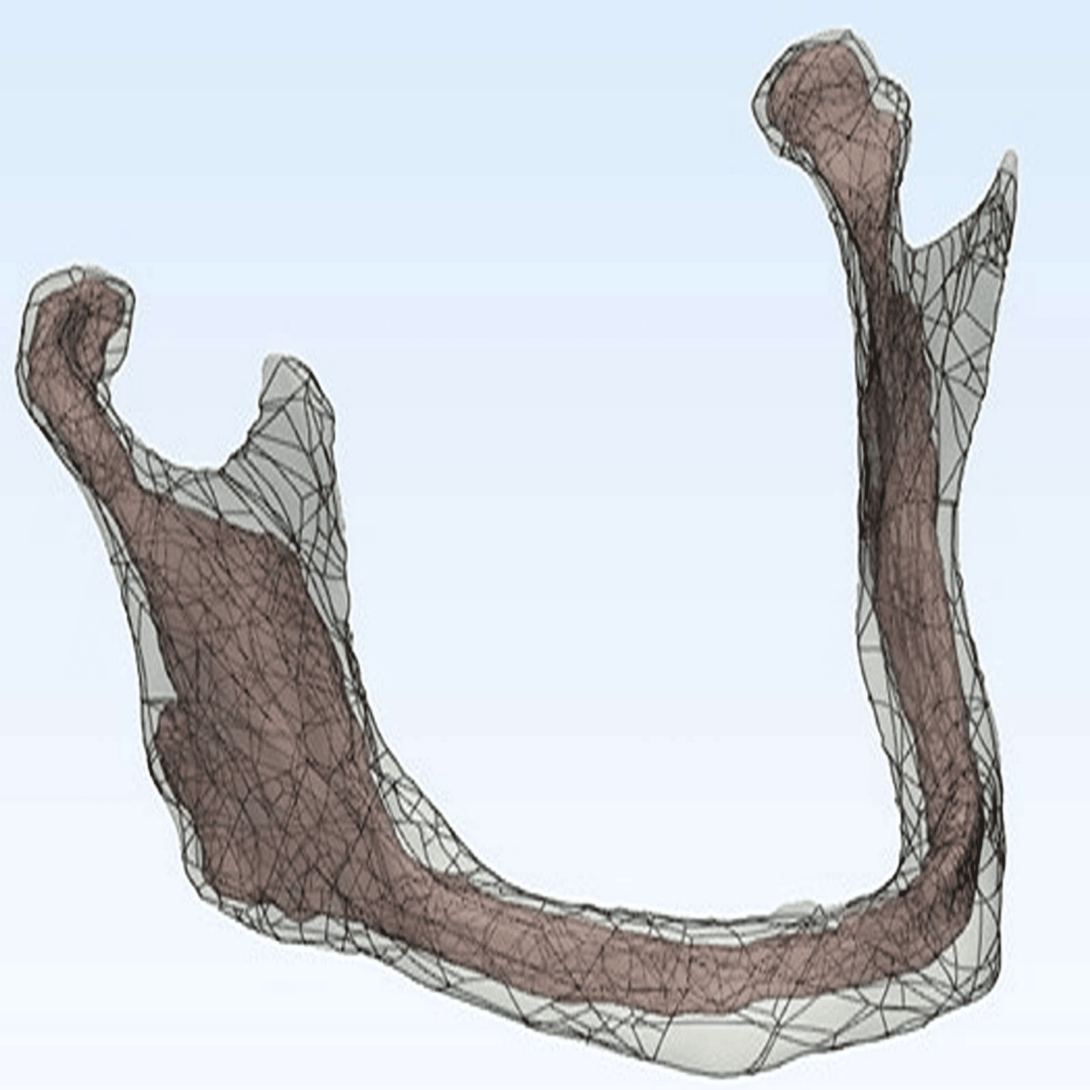
Dividing surfaces of the mandibular model into patches using Materialise 3-matic software Materialise 3-matic software: Materialise N.V., Leuven, Belgium.

The study sample included a 3D model of a fully edentulous mandible obtained from a cone beam computed tomography (CBCT) image of a 55-year-old male edentulous patient who was referred to the Department of Removable Prosthodontics at the Faculty of Dentistry, Damascus University. The 3D model of the mandible in this study was constructed in a manner similar to the studies of Sirandoni et al. [8] and Durkan et al. [9] using a CBCT image of the mandible. Models of the cortical and cancellous bone were constructed in the form of 3D surface structures and exported in STL file format using Mimics software (Materialise’s Interactive Medical Image) from (Materialise N.V., Leuven, Belgium) company. The mesh structure of the surfaces was smoothed and simplified, and errors in this structure were detected and corrected. After that, the surface of each component was divided into quadrilateral surfaces called patches using Materialise 3-matic software (Materialise 3-Matic Medical) from (Materialise N.V., Leuven, Belgium) company (Figure 1).

**Figure 2:**
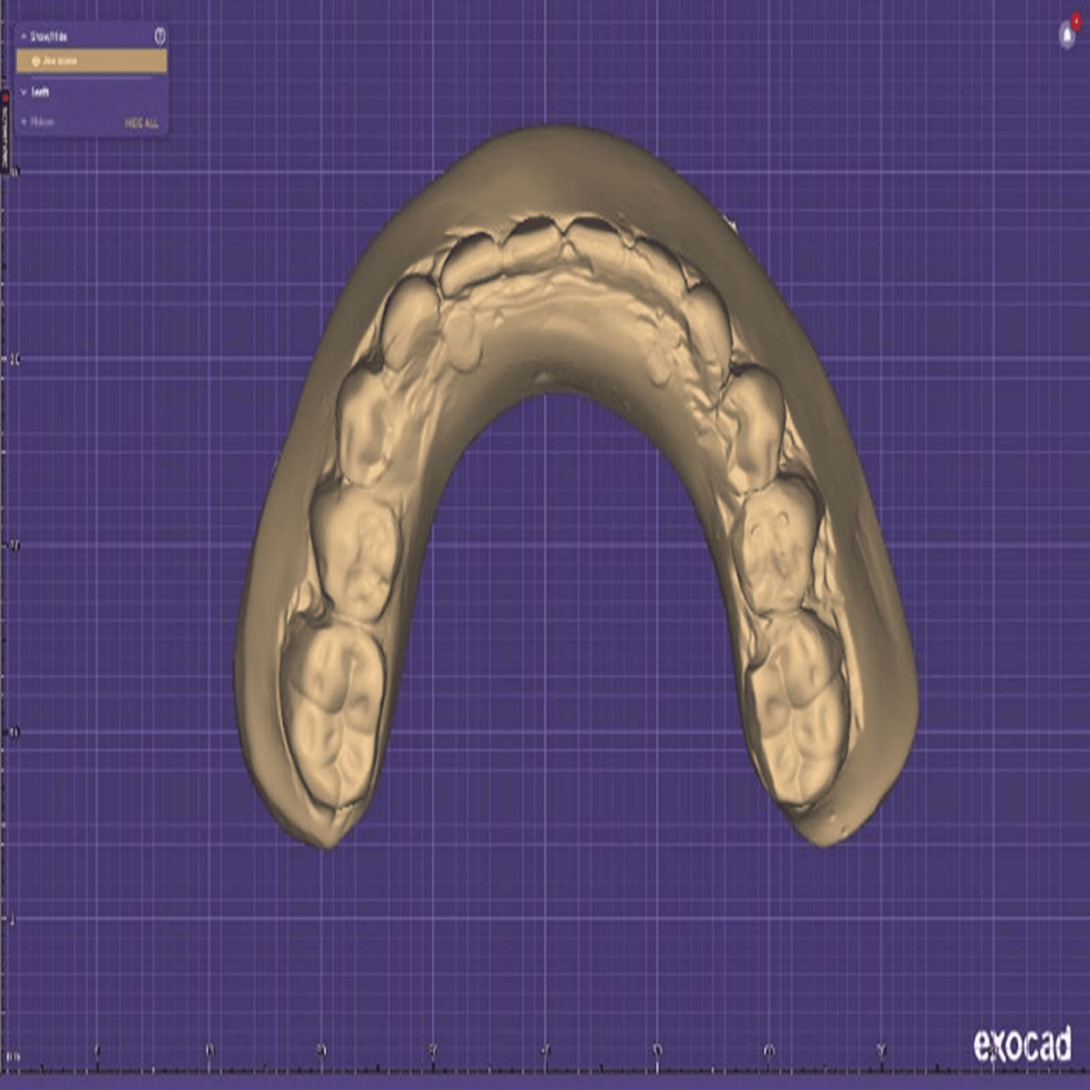
Digital scan of acrylic prosthesis in STL file format STL: Stereolithrography.

An acrylic prosthesis was fabricated for the patient, and a digital scan was performed using a MEDIT T300 office scanner (Medit Corp., Seoul, Korea) (Figure 2).

**Figure 3:**
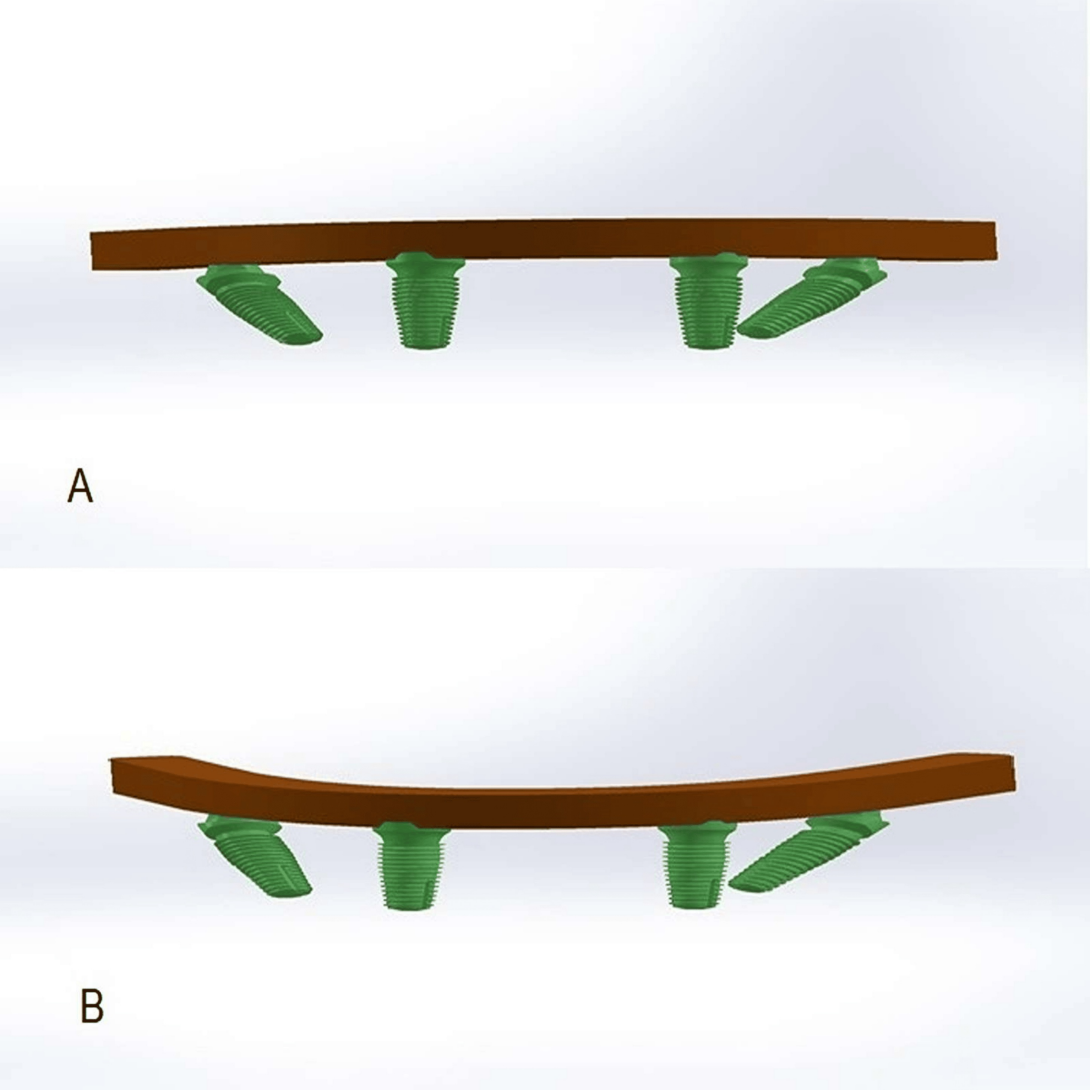
The constructed frameworks A-Framework with 10 mm cantilever length with abutments and implants. B-Framework with 15 mm cantilever length with abutments and implants.

SOLIDWORKS software (2021) (Dassault Systèmes SolidWorks Corporation, Waltham, MA) was used to construct frameworks and, implants and abutments. A digital model of the implants and abutments was obtained from MegaGen company (Seoul, Korea). The implant diameter was 4 mm and the length was 11.5 mm. Four implants were placed in the model: two anterior implants were vertical and located between the lateral incisor and the canine on each side, and two posterior implants were placed with a distal tilt of implants 30 degrees [9,10]. Frameworks were designed to fit the prosthesis as a solid geometric. The height was 6 mm, the width was 5 mm, and the geometric shape was a horseshoe, considering that shape is consistent with the shape of the mandible [10]; cantilever lengths were 10 and 15 mm (Figure 3).

**Figure 4:**
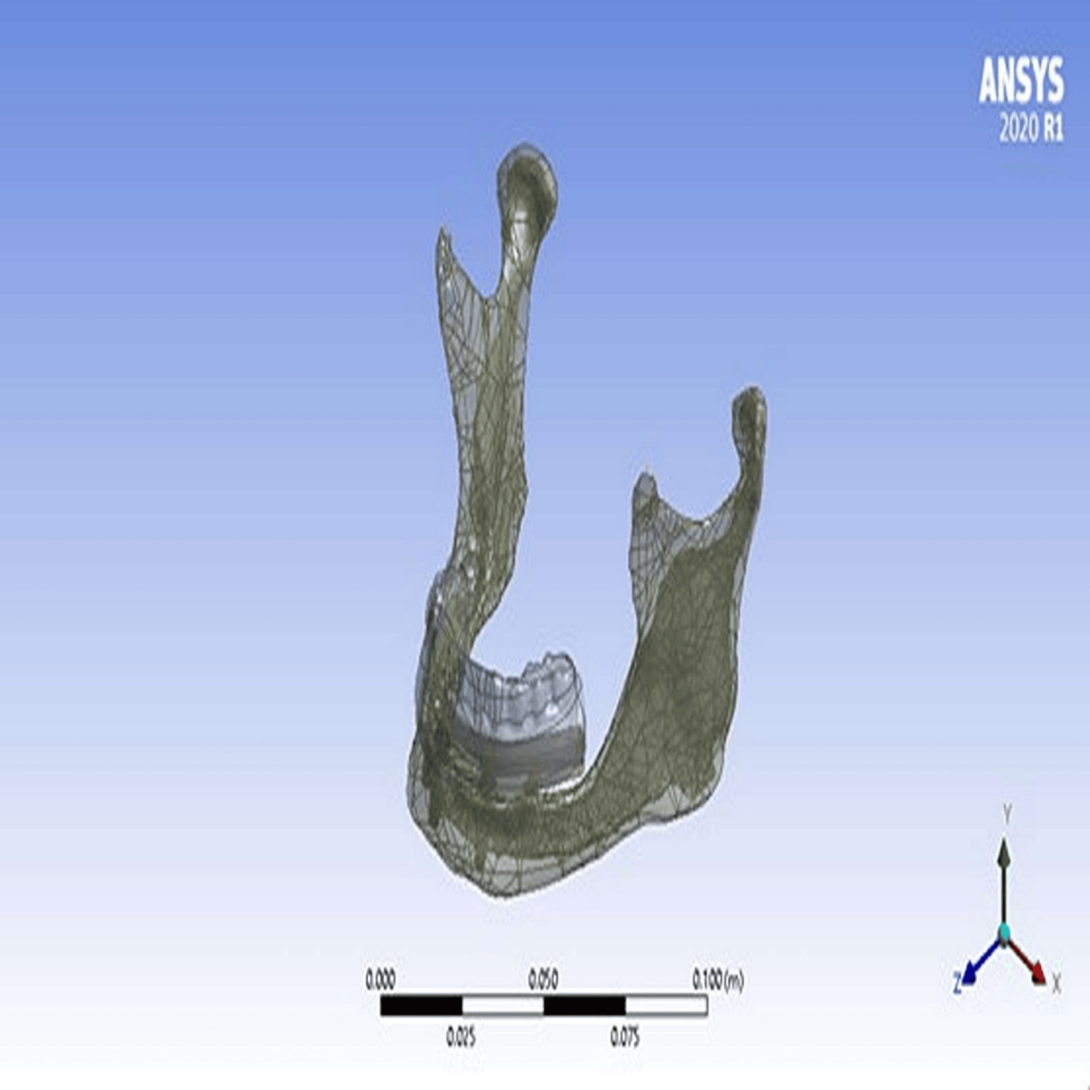
Mandibular model with implants, abutments, framework and acrylic prosthesis in Ansys software Ansys (Ansys, Canonsburg, PA).

Figure 4 shows a model of a mandible with implants and abutments that connect frameworks with cantilever lengths 10 and 15 mm, and the acrylic prosthesis. The final model was saved after exploring and correcting minor errors in Parasolid format, which can be exported and processed within the three-dimensional finite element analysis program Ansys (Ansys, Canonsburg, PA).

The simulation was performed using Ansys software (R2 2020). A static structural solver was used because the analysis to be performed was static. Material properties were determined using the modulus of elasticity and Poisson's ratio for each material (Table 1).

**Table 1:** : Material properties

Material	Modulus of Elasticity	Ratio Poisson's
Cortical bone	13	0.3
Spongy bone	1.4	0.3
Titanium	102	0.35
Acrylic	2.7	0.35
PEEK	4.2	0.36

**Figure 5:**
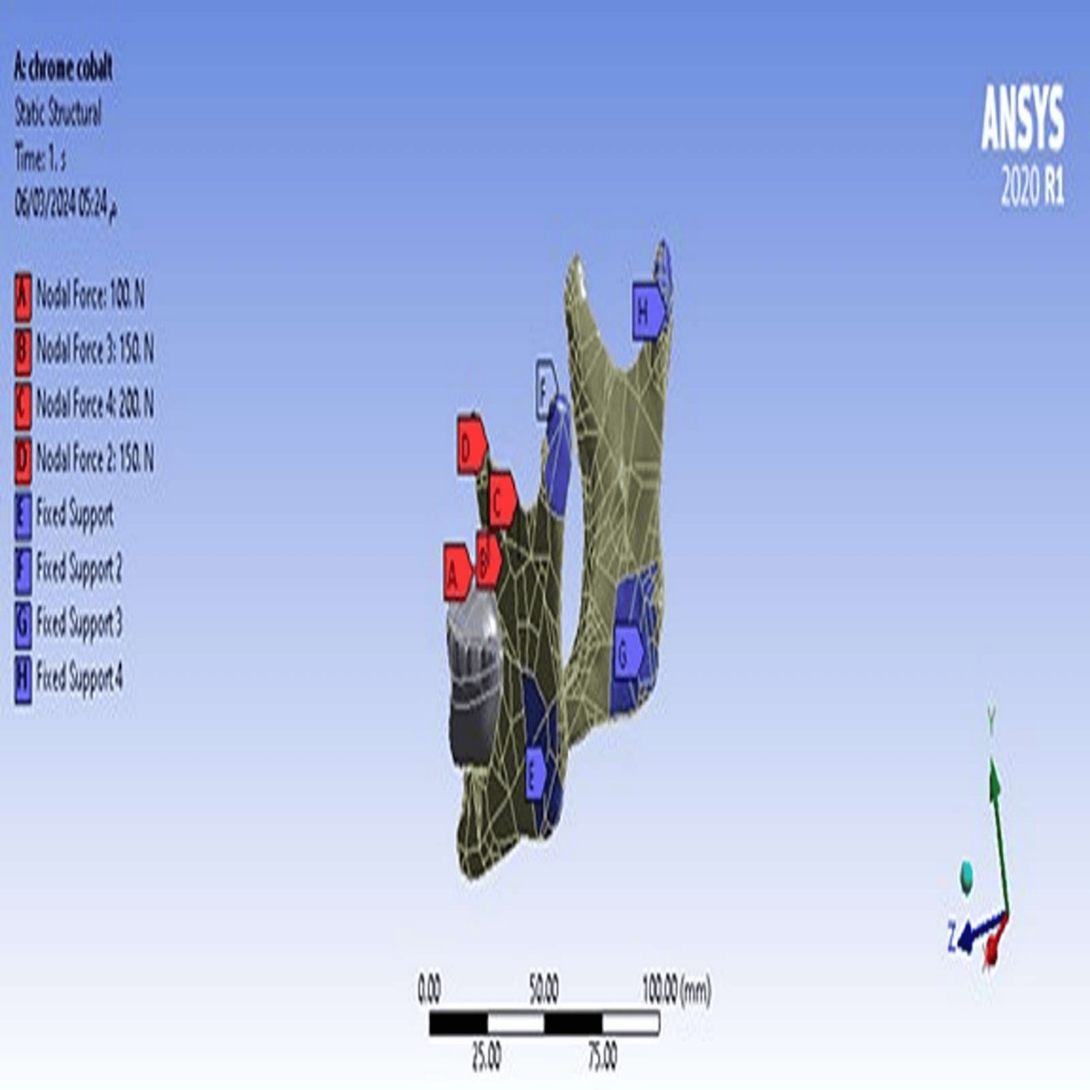
Boundary conditions

The geometric shape to be studied (mandible) and the materials it consists of were defined. The contact interface between parts of the model was defined as being in a state of total conformity. Boundary conditions of model fixation areas were determined by fixing the anchor of each of the two masseter muscles, two medial pterygoid muscles, and mandibular condyles. Muscle anchors were determined using the literature reference [11] (Figure 5).

**Figure 6:**
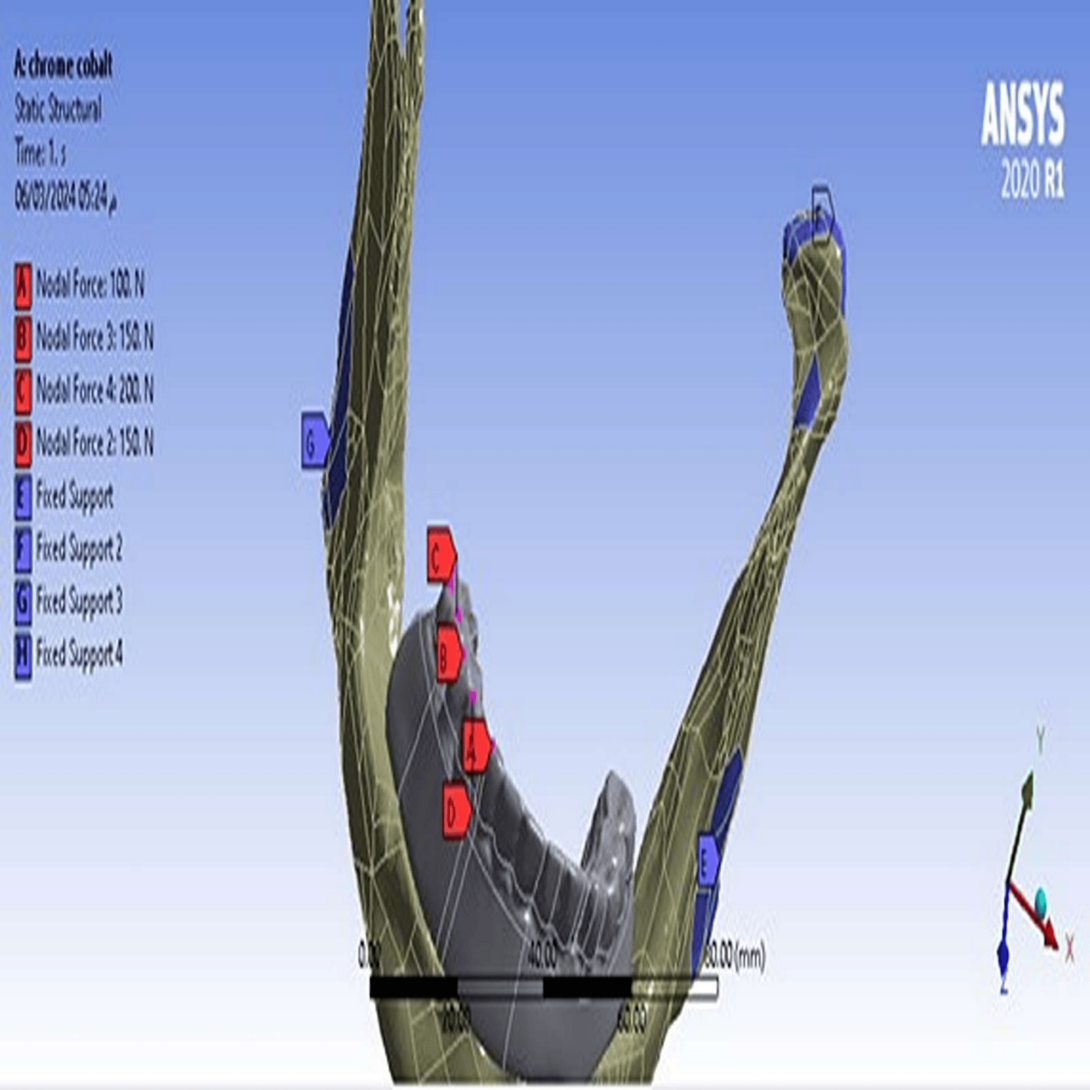
Forces and loads distribution locations

Occlusal forces were applied from the right side at a 45-degree angle totaling 600 N: 100 N at the right canine cusp peak, 150 N at the first premolar buccal cusp peak, 150 N at the second premolar buccal cusp peak, and 200 N at the first molar mesio-buccal and disto-buccal cusps peak [9] (Figure 6).

The model was then divided into 1,245,655 elements, and stresses to be studied in important areas of the model were determined before conducting the finite element analysis. In order to achieve the aim of this study, the maximum principal stress (tensile stress) and the minimum principal stress (compressive stress) were studied in the cortical bone and spongy bone, and the equivalent stress (Von Mises Stress) in implants and frameworks as given in the studies by Ozan et al. [2], Durkan et al. [9] and Haroun and Ozan [12]. A study group was created for each cantilever length, and the prosthetic framework material was PEEK in both groups. Results were shown in the form of color distribution and numerical values ​​to express the distribution of stresses in each model.

## Results

The maximum and minimum principal stress values ​​in the cortical bone

**Figure 7:**
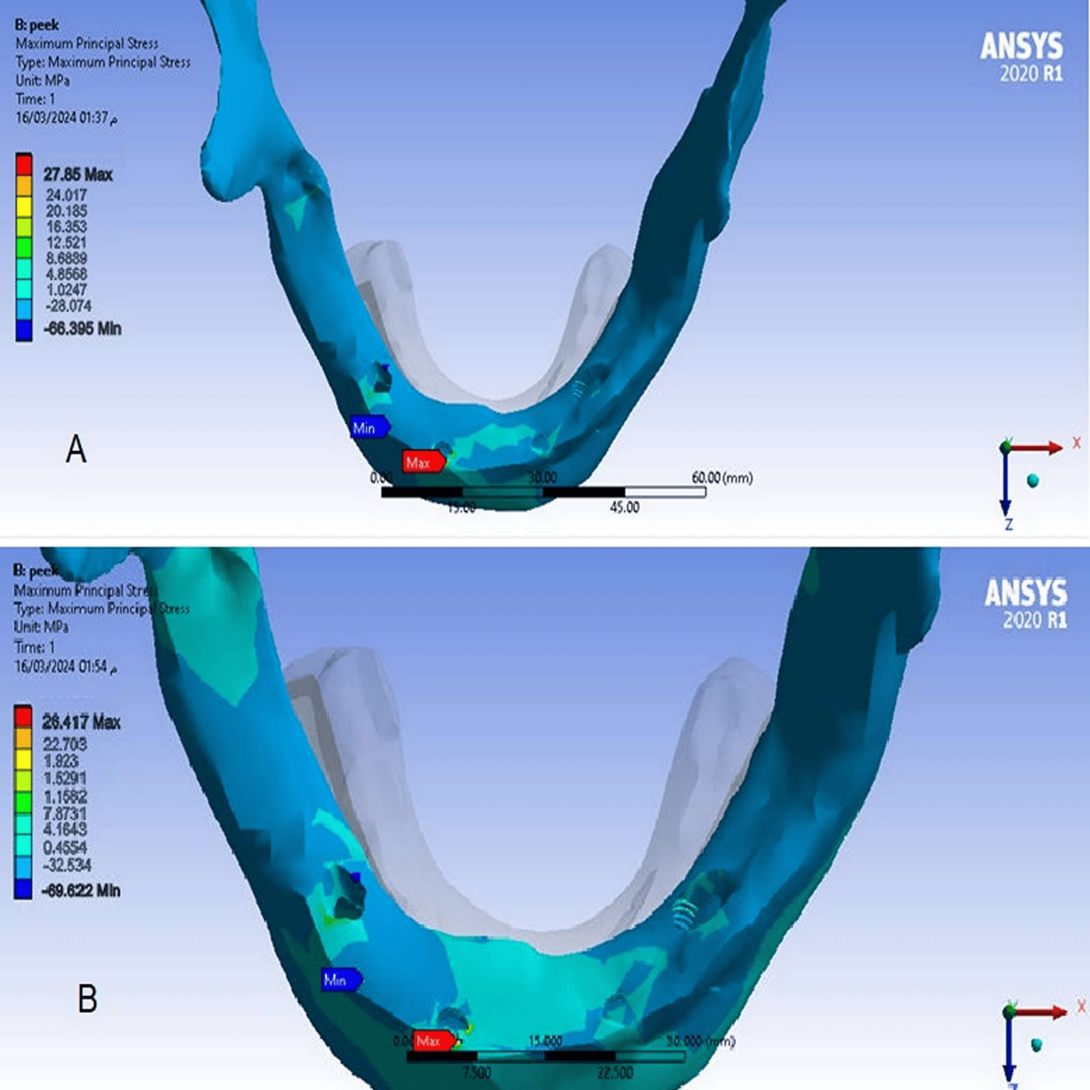
The maximum principal stress in the cortical bone A-At a cantilever length of 10 mm, B-At a cantilever length of 15 mm.

**Figure 8:**
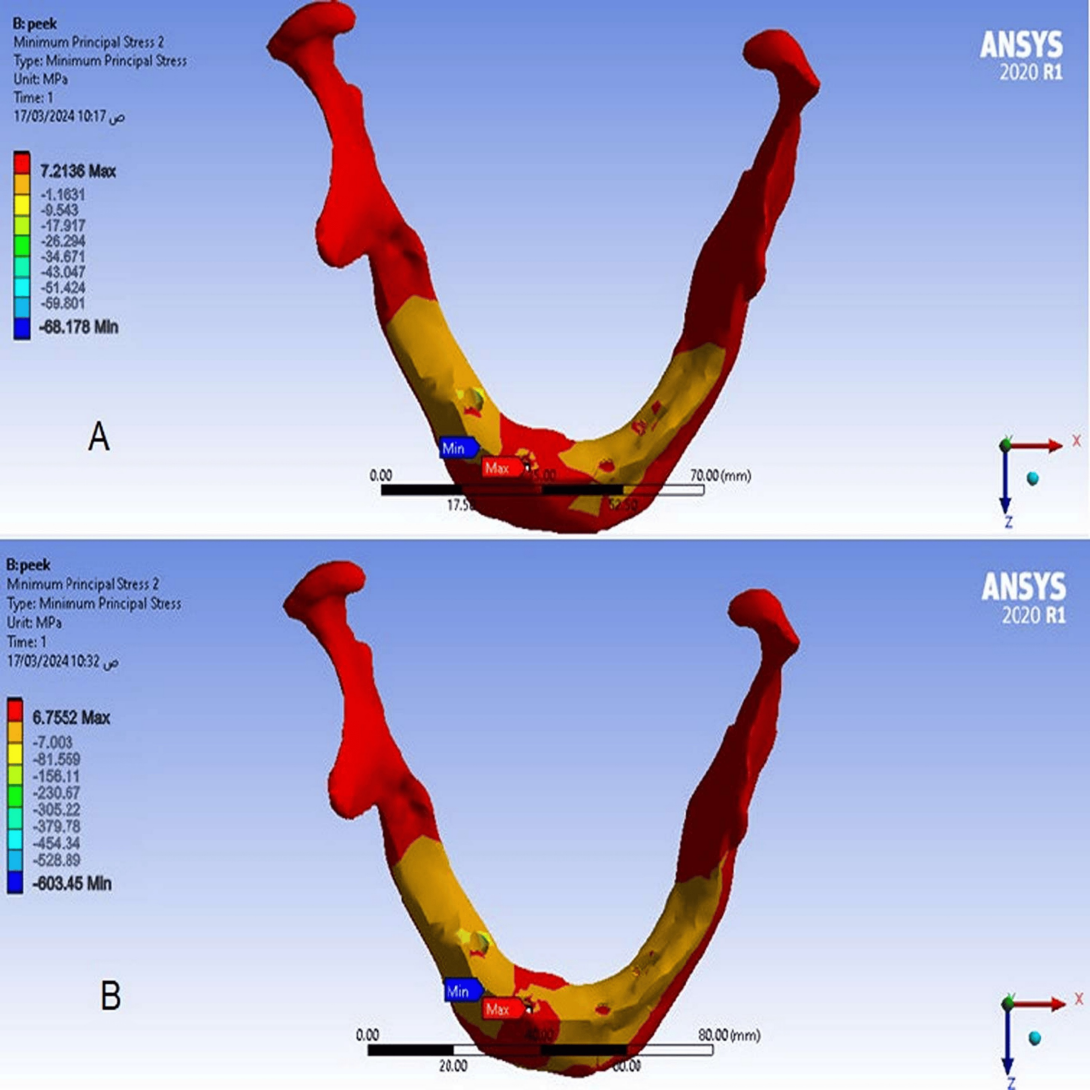
The minimum principal stress in the cortical bone A-At a cantilever length of 10 mm, B-At a cantilever length of 15 mm.

The maximum and minimum principal stress values ​​in the cortical bone at a cantilever length of 15 mm (Model 1) were slightly decreased compared to stress values ​​when the cantilever length was 10 mm (Model 2), and the maximum stress value was concentrated at the right anterior implant (Figures 7, 8).

The maximum and minimum principal stress values in the spongy bone

**Figure 9:**
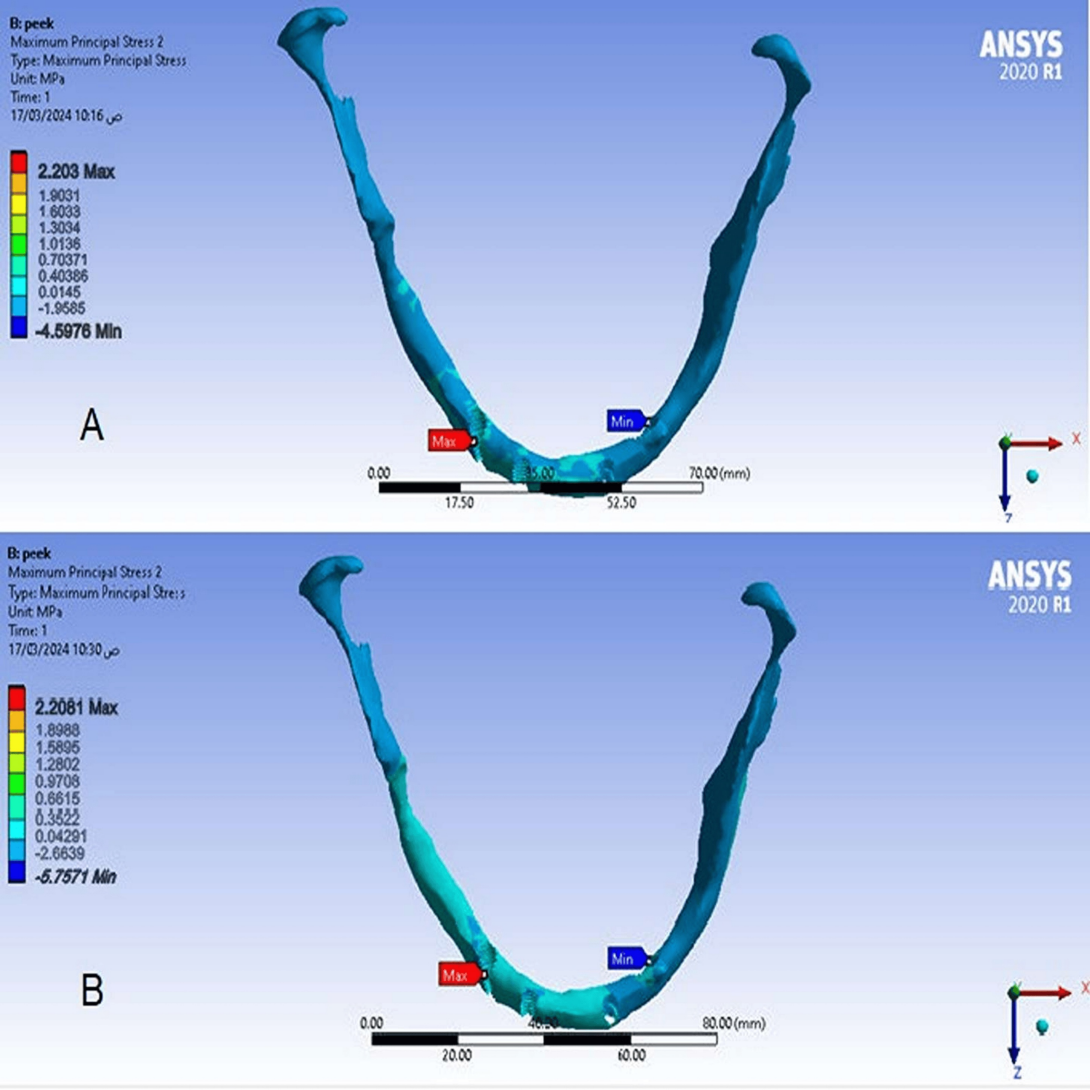
The maximum principal stress in the spongy bone A-At a cantilever length of 10 mm, B-At a cantilever length of 15 mm.

**Figure 10:**
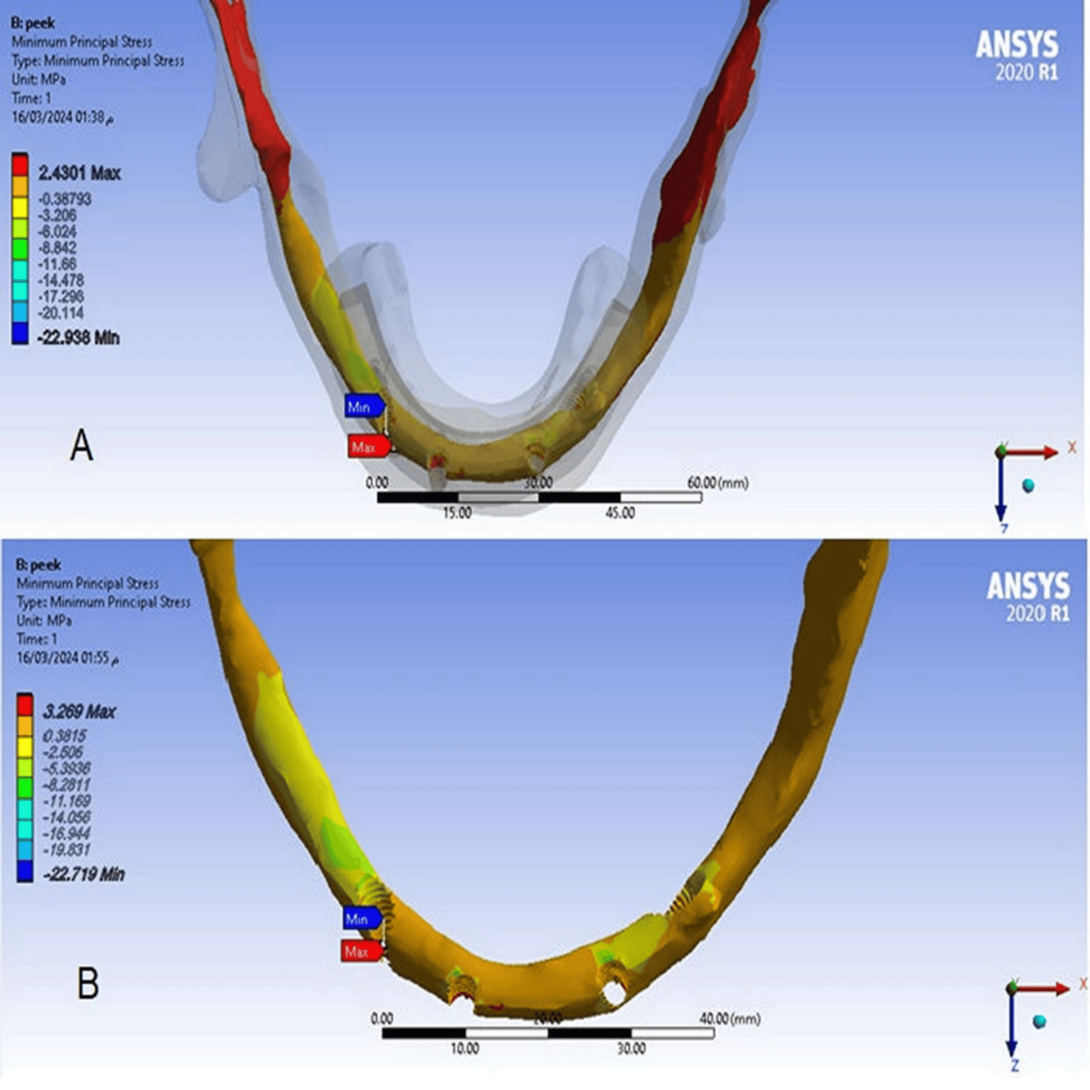
The minimum principal stress in the spongy bone A-At a cantilever length of 10 mm, B-At a cantilever length of 15 mm.

There was no difference between values ​​of maximum and minimum principal stresses in the spongy bone at a cantilever length of 15 mm model (1), and at a cantilever length of 10 mm model (2), and maximum stress values ​​were concentrated at the right posterior implant (Figures 9, 10).

The Von Mises stress values in implants

**Figure 11:**
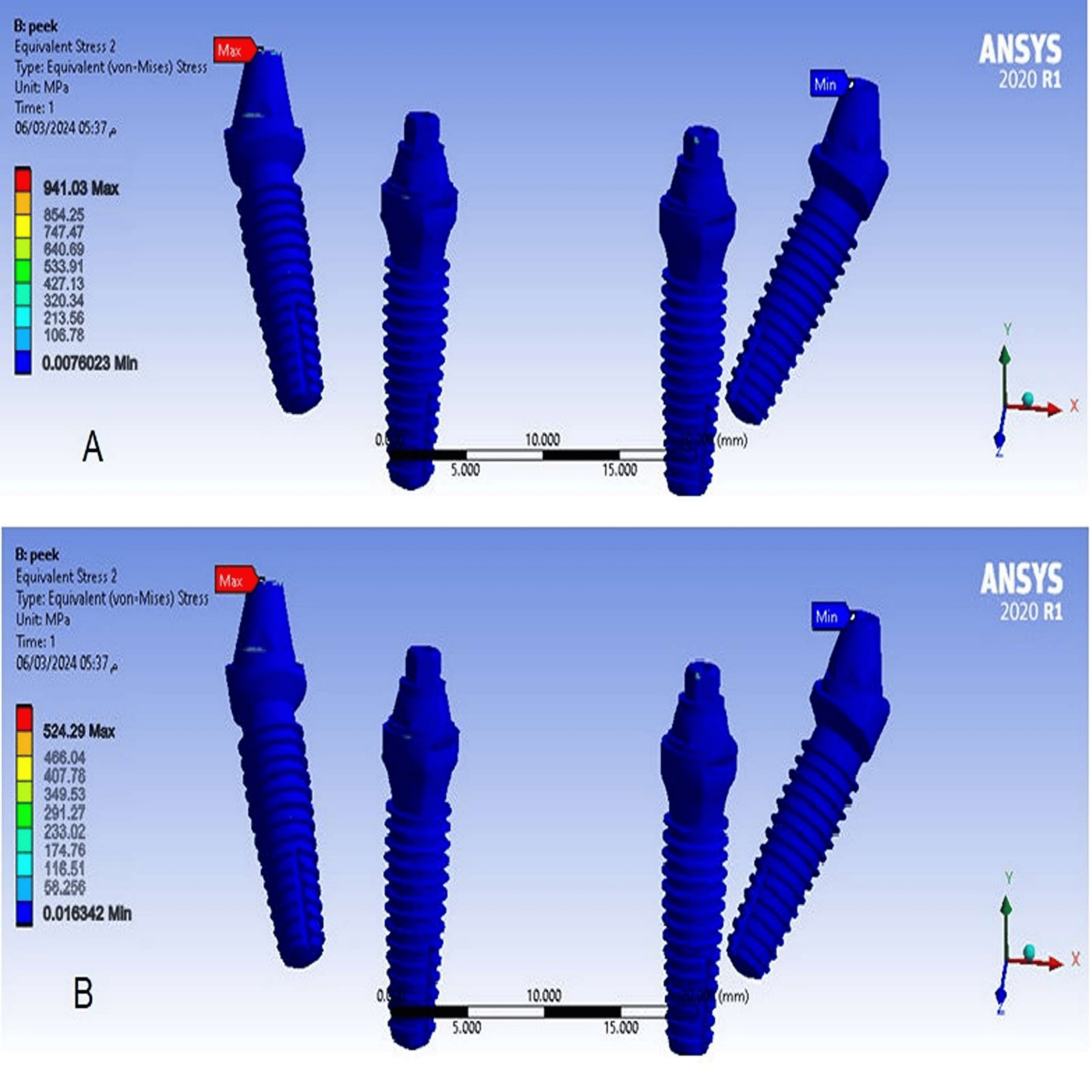
The Von Mises stress in implants A-At a cantilever length of 10 mm, B-At a cantilever length of 15 mm.

Von Mises stress values ​​in implants decreased significantly when the distal cantilever length was 15 mm (model 1), compared to stress values ​​at a cantilever length of 10 mm (model 2), and maximum Von Mises stress values ​​were concentrated at the right posterior implant abutment (Figure 11).

The Von Mises stress values in frameworks

**Figure 12:**
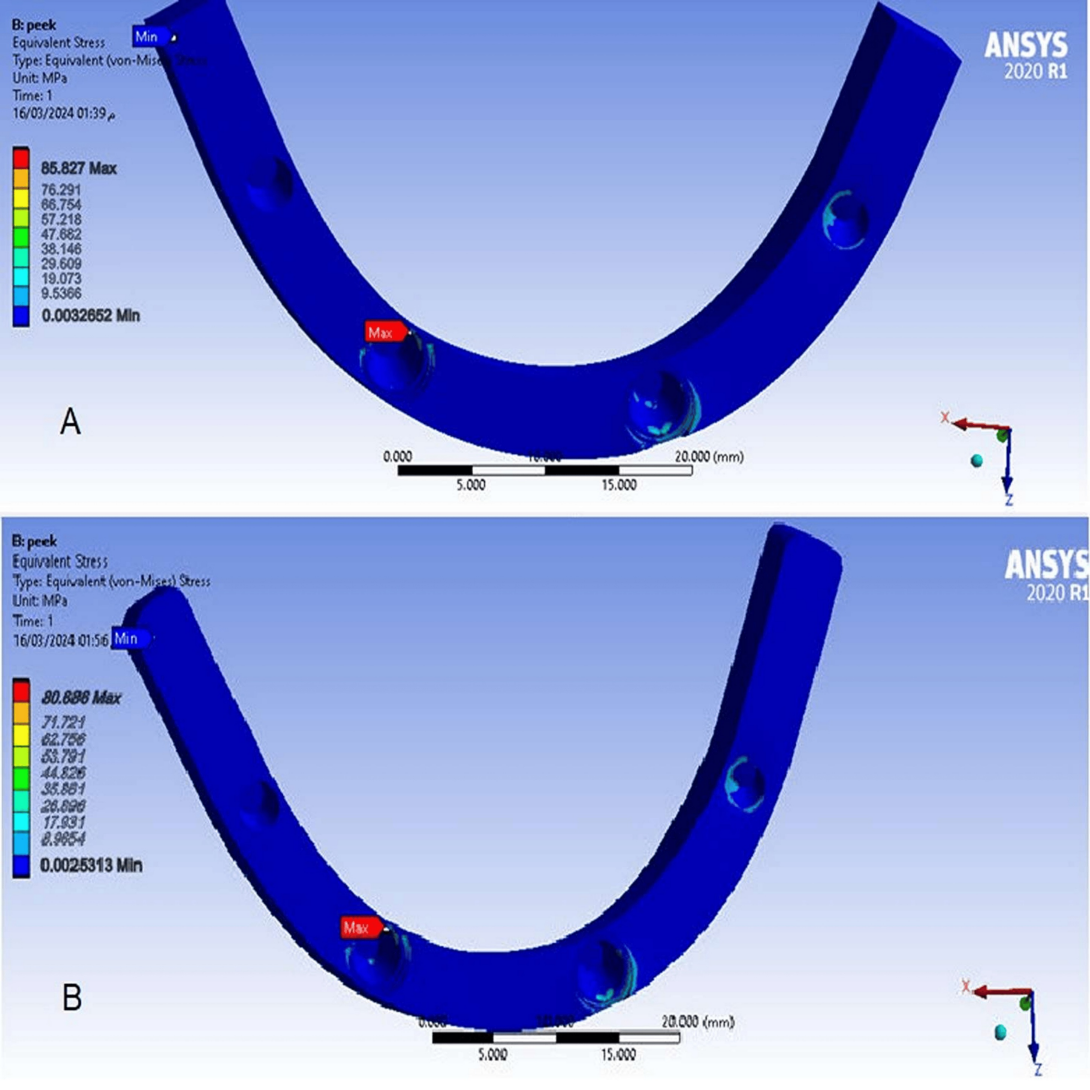
The Von Mises stress in frameworks A-At a cantilever length of 10 mm, B-At a cantilever length of 15 mm.

Von Mises stress values ​​in a framework with a cantilever length of 10 mm (Model 2) showed higher values ​​compared to Von Mises stress values ​​in a framework with a cantilever length of 15 mm (Model 1). Maximum Von Mises stress values ​​in frameworks were concentrated at the location of engagement of frameworks to the left anterior implant abutment (Figure 12).

Von Mises stress values ​​in frameworks in models were higher than the ultimate stress of the PEEK material, which led to the deformation of the prosthetic framework material (Table 2).

**Table 2:** Stresses values of models in cortical and spongy bone, implants, frameworks

PEEK		
CL 15 mm Model (1)	CL 10 mm Model (2)	
26.417	27.85	Cortical (MPa) Max
6.7552	7.2136	Cortical (MPa) Min
2.2081	2.203	Cancellous (MPa) Max
3.269	2.4301	Cancellous (MPa) Min
524.29	941.03	Implants (MPa) VM
80.686	85.827	Frameworks (MPa) VM

## Discussion

The use of implant-supported fixed prostheses for the complete dental arch is a treatment option for oral rehabilitation in patients with complete teeth loss [13,14]. However, due to the anatomical constraints of the edentulous mandible or the limited quality and quantity of the posterior alveolar bone, implant-supported prosthetic treatment is impossible without complex surgical interventions prior to implant placement [14,15]. The concept “All-on-4” was invented by Maló in 2003 as a method that allows physicians to overcome anatomical constraints of the mandible without the need for advanced and risky surgical techniques [14,16,17]. Metals and zirconia have been widely used to make the frameworks for these prostheses due to their high mechanical properties and biocompatibility [18-20]. However, high stress in the bone has been reported when using non-polymeric or solid frameworks with high elastic modulus, so PEEK has been presented as a promising material for making implant-supported prostheses with shock-absorbing properties and an elastic modulus close to that of bone [8,18,21], but so far it has not been widely approved clinically due to limited research and little scientific evidence on the effectiveness of this material [18,22].

A cantilever is often needed from the posterior implants in this type of prosthesis, and the presence of a distal cantilever will increase the load and stress distributed on the implants [23]. There are few studies on the length of the distal cantilever in the All-on-4 technique [24]. Prosthetic frameworks were designed, and their geometric shape was horseshoe, considering that this shape is compatible with the shape of the mandible [10]. The lengths of the distal cantilever in frameworks were 10 and 15 mm. It is necessary that the cantilever length does not extend more than twice the AP distance or to more than 20 mm, because it will increase the stress and load applied to the implants [25]. Posterior implants were placed at a distal tilt of 30°, as many studies have found that this is the most suitable angle for tilt compared to other angles (15 and 45) degrees [16]. The boundary conditions in biomechanical studies when using the finite element method were simple in the past, such as restricting the model from the edge of the mandible [26], but in this study, a semi-realistic simulation of the masticatory muscles was conducted by determining the anchor surface of each part of the masseter and medial pterygoid muscles on both sides, and the mandibular condyles based on the anatomical reference [11], and then the direction and strength of each muscle were determined by three components in the spatial axes (X_Y_Z), and these components were obtained from the Cruz et al.'s study [27]. In this study, occlusal forces were applied from the right side at a 45 degree [9] because it is very rare for the forces applied to the implants to be directed directly according to the longitudinal axis of implants only. When studying an occlusal scheme for an implant-supported prosthesis, we will find large amounts of forces applied directly according to the buccal-lingual axis (lateral loading) [7]. The total forces were 600 N, similar to the bite forces in normal humans, and the forces were distributed as follows in order to simulate the clinical reality more: 100 N at the right canine cusp peak, 150 N at first premolar buccal cusp peak, 150 N at second premolar buccal cusp peak, and 200 N at first molar mesio-buccal and disto-buccal cusps peak [9].

Values ​​of maximum and minimum principal stresses in the cortical bone

Values Decreased at a cantilever length of 15 mm, which is in contrast to the study of Durkan et al. [9], which found that the stress values ​​in the cortical bone were higher in the Zr model with a longer cantilever. This decrease may be due to the fact that increasing cantilever length in the PEEK material led to an increase in the mass of the material and the length of the polymer chains, thus increasing the material’s expansion and better dissipation of stresses. We also found that the highest values ​​of the maximum and minimum principal stresses were in the cortical bone surrounding the right anterior implant, due to the difference in bone quality between the anterior and posterior regions of the mandible, as the anterior region usually has a greater amount of cortical bone than spongy bone.

Values of maximum and minimum principal stresses in the spongy bone

This study found no difference in values ​​of the maximum and minimum principal stresses in the spongy bone in models because the elastic modulus of PEEK is very close to the elastic modulus of spongy bone. This study also agreed with the result of Sirandoni et al. [8] that the highest values ​​of the maximum and minimum principal stresses in the spongy bone were in the spongy bone surrounding the right posterior implant. This may also be attributed to the difference in bone quality between the anterior and posterior regions of the mandible.

Values of Von Mises stress in implants

Model 1 at a cantilever length of 15 mm showed a significant decrease in the Von Mises stress value in the implants compared to model 2 at a cantilever length of 10 mm because increasing the cantilever length led to an increase in the mass of the elastic material and thus an increase in the length of the polymer chains, making them more extensible and better at dissipating stresses. This study also found that the highest equivalent stress values ​​were concentrated at the right posterior implant abutment in models, which is consistent with studies of Durkan et al. [9], Haroun et al. [12], and Bhering et al. [20].

Values of Mises stress in frameworks

Model 1 at 15 mm cantilever length showed a lower Von Mises stress value in framework than Model 2 at 10 mm cantilever length, but these values ​​exceeded the ultimate stress of PEEK (80 MPa), and there was deformation in the framework material [8,28]. The highest equivalent stress value in the framework was concentrated in the area of ​​the framework’s connection to the abutment cervically in all models [8,20]. The highest value was located at the left anterior implants due to the flexibility and expansion of the material when forces are applied, and thus the highest stress value will move to a location farther away from the force application (Right side). One of the biggest limitations of this study is that it is limited to the biomechanical aspect only and the application of unilateral oblique occlusal forces as well.

## Conclusions

This study found that increasing the distal cantilever length in the PEEK framework reduced stress values ​​in cortical bone, implants, and prosthetic framework. Results of this study showed that stress values ​​within frameworks exceeded the ultimate stress of the PEEK material and deformation of the prosthetic framework material occurred. Although biomechanical studies are performed under ideal conditions, finite element studies can be useful in providing a prior prediction of results before clinical research is conducted. Therefore, within the limits and conditions of this study, we recommend not using PEEK in the construction of frameworks in implant-supported prostheses according to the All-on-4 technology, and replacing it with materials with a high modulus of elasticity.
